# Normabaric Hyperoxia Treatment Improved Locomotor Activity of C57BL/6J Mice through Enhancing Dopamine Genes Following Fluid-Percussion Injury in Striatum

**Published:** 2013-12

**Authors:** Sangu Muthuraju, Syed Taha, Soumya Pati, Mohamed Rafique, Hasnan Jaafar, Jafri Malin Abdullah

**Affiliations:** 1 Department of Neurosciences, School of Medical Sciences, Universiti Sains Malaysia, 16150 Kota Bharu, Kelantan, Malaysia;; 2 Department of Pathology, School of Medical Sciences, Universiti Sains Malaysia, 16150 Kota Bharu, Kelantan, Malaysia

**Keywords:** Fluid-Percussion Injury, Closed Traumatic Brain Injury, IntelliCage, Locomotor activity and Normabaric hyperoxia

## Abstract

Closed traumatic brain injury (CTBI) leads to increase mortality rates in developing countries. However, a sustainable therapeutic approach has not been established yet. Therefore, the present study was designed to evaluate the impact of normabaric hyperoxia treatment (NBOT) on striatum associated Locomotor Activity (LA) in IntelliCage after Fluid-Percussion Injury (FPI). Animals were divided in four groups: Group I control (n=24), Group II sham (n=24), Group III FPI (n=24) and Group IV FPI with NBOT (n=24). Animals were habituated in IntelliCage for 4 days following transponder implanted in mice neck region on day 5. Then the LA of all groups was assessed 6hr daily for 5 days before inducing FPI. On day 6, cannula was implanted on the striatum, on day 7 FPI was performed in Group III (kept in normal environment) and IV (immediately exposed to NBOT for 3 hr). LA (in terms of number of visits in all four corners) was assessed 6 hr at days 1, 7, 14, 21 and 28 following FPI. After the animals were sacrificed to study the neuronal damage, dopamine receptors and transporters expression in striatum. The results suggested that the LA of FPI impaired mice as compared to the control and sham showed less number of visits in all four corners in IntelliCage. Morphological results revealed that FPI induced neuronal damage as compared to sham and control. Dopamine receptors and transporters were down regulated in the FPI group as compared to the control. Immediate exposure to NBOT improved LA in terms of increased number of visits in all four corners, reduced number of cell death and improved receptor expression as compared to FPI. In conclusion, NBOT exposure could improve the LA of mice following FPI through prevention of neuronal damage, improved dopamine receptors and transporters.

## INTRODUCTION

Victims of traumatic brain injury (TBI) suffer short and long term physical and behavioral impairments that depend on the severity of the injury (1). TBI is considered as a heterogeneous condition divided into acute, sub-acute and chronic pathologies (43). TBI has been studied in the laboratory using several experimental models (21). Many animal models have been introduced to produce brain injury, such as fluid percussion (FP) (7, 44, 46), providing insight into the cellular and mechanical mechanisms of central nervous system (CNS) dysfunction and cell death. Some of them, like the FP model, produce a direct trauma to the exposed brain which produces a closed-head TBI (CTBI) (22). Most of the studies suggested that the applied brain injuries are severe enough to produce edema, a break in the blood-brain barrier (BBB) and induce morphologically evident brain damage (5). In mice and rats, a severe TBI results in a local lesion cavity followed by evolving necrotic and apoptotic cell death processes accompanied by persistent motor impairment. (12, 36).

However, a sustainable therapeutic approach has not been established yet. Several brain regions are affected by TBI including but not limited to the hippocampus, frontal cortex and striatum (3, 26, 30). These three regions are important because of their role in attention, executive function, learning and motor functions (48). Among these, striatum is one of the most important brain regions involved in higher-level organizational aspects of learning in human (42). This is also a crucial element in the neural circuitry underlying motor control (34). Most of studies reported that TBI causes selective hippocampal cell death, which is believed to be associated with cognitive impairment observed in clinical and experimental settings (2, 37) and TBI induced dysfunction of the prefrontal cortex causes many high level cognitive deficits including memory dysfunction (14, 17). A number of research groups have attempted to develop novel and innovative protocols to replicate closed traumatic brain injuries in animal models, but few experimental models have been successful (13, 27, 28).

But a few studies investigated the effect of TBI on striatum associated locomotor activity of mice. However, tissue damage after TBI is not limited to discrete brain regions. Diffuse axonal injury in white matter tracts along with gray matter damage further complicates the clinical presentation of brain injury. The widespread disruption of neuronal projections has implications for all neurotransmitter systems, including DA. Dopamine, a major neurotransmitter in the mammalian CNS, is involved in the control of locomotor activity and pathways regulating goal-oriented behavior. Disturbances in dopamine neurotransmission contribute to locomotor activity dysfunction after TBI. The changes in dopamine neurotransmission may be mediated by alterations in the dopamine transporter, which plays a key role in maintaining dopamine homeostasis (40). Disruption in dopamine (DA) neurotransmission in basal ganglia is believed to be one of the underlying factors for cognitive deficits and motor impairment following severe TBI insult (4). Neurochemical studies also suggest that there are alterations in the levels of striatal dopamine and proteins that synthesize and transport dopamine after injury (4, 49). In brain areas known to be damaged in TBI, DA receptors are seen to be significantly expressed, and dopaminergic dysfunction after TBI is believed to be regulated by dopamine transporter (DAT), dopamine receptor 1 (DR1) and dopamine receptor 2 ( DR2) genes (4). DA signaling dominates in basal ganglia (8) and regulated by glutamate input to striatum (25). Central dopaminergic system in striatum is vulnerable to injury (24) and disruption in DA neurotransmission following TBI may be mediated by alterations in the dopamine transporter, as shown by a Western blot study in trauma-induced brain injury in the rat frontal cortex (50).

Nowadays, the most common TBI management includes monitoring intracranial pressure (ICP), followed by treatment to reduce ICP immediately after the TBI insult. It is believed that secondary neuronal damage might be prevented by reducing ICP using a therapeutic approach. Since therapeutic tools have not been established to reduce the neuronal damage through reduced ICP and increased CBF following TBI, it is assumed that the most potential therapeutic interventions for TBI are to reduce ICP and increase CBF, as well as improving brain oxygenation through immediate exposure to NBOT after TBI insult. NBOT treatment involves the administration of 100% oxygen in the first hours after a TBI insult. Therefore in the present study we hypothesized that the immediate exposure to normabaric hyperoxia for 3h could be a possible therapeutic approach for improving locomotor activity through reduced neuronal damage and increased dopamine genes in striatum for TBI in the mice model.

## MATERIALS AND METHODS

### Chemicals

The following chemicals were purchased from sigma such as Ketamine, Xylazine, Paraformaldehyde, Ethanol, Hematoxylin and eosin, Xylene, DPX, Triton andBovine Serum Albumin (BSA). FITC-Annexin apoptotic kit (Abcam), Panneuronal marking (Millipore) and antibodies for DR1, DR2, DAT and VMAT(Invitrogen) were used for neuronal damage and Immunohistochemistry.

### Subjects

All experimental procedures on animals were first approved by the Animal Ethical Committee, Universiti Sains Malaysia, as outlined in the NIH Guide for the Care and Use of Laboratory Animals. These studies used a minimum number of subject animals, and appropriate procedures were used to minimize the possibility of pain and suffering. This study was used ninety six C57BL/J6 male mice weighting between 25 and 30 g (mean 28 g) maintained by the Animal Research and Service Centre (ARASC), Universiti Sains Malaysia. Prior to any behavioral and surgical procedures, mice were housed individually in standard Plexiglas housing cages and maintained at 22°C on a 12 h light/dark cycle with food and water available ad libitum.

### Experimental Groups

The mice were randomly divided in to four groups. The (i) Control (n=24) and (ii) Sham group (n=24) were anesthetized and connected to the injury device, without releasing the pendulum. The (iii) Fluid-Percussion Injury (FPI) group (n=24) were administered injury without exposure to Normabaric hyperoxia (NBO). The (iv) FPI group (n=24) were administered injury, followed by immediate exposure to NBO for 3 h. Twelve animals of each group were used for behavior studies and six animals for studying the morphological changes, apoptotic neuronal damage, six animals for pan neuronal marking and Immunohistochemistry following behavior study.

### Surgery

Adult C57BL/J6 male mice weighting between 25 and 30 g were surgically prepared under Ketamine + Xylazine (2.6/0.16 mg per animal). Animals were mounted on a Stereotaxic frame secured by incisor bar and ear bars. Mechanical ventilation maintained anesthesia with 2% isoflurane in 2:1 N2O/O2 and syringe hub placed 0.2 mm anterior and 2.3 mm lateral to the bregma. Dental acrylic was then poured about the syringe hub and the animal was returned to its home cage. The sham group received only syringe hub.

### Induction of FPI injury

TBI was induced on the mouse using Fluid-Percussion Device (FPD) (Dragonfly Inc., Model HPD 1700, Virginia, and USA) with modified method of (5, 23). Mice were connected to the FPI device with saline filled high pressure tubing. A pulse of 21-23 msec with 3.6 ± 0.1 atm (5) was rapidly injected into the cranial cavity. The strike from the pendulum can be calibrated using a transducer that connects to a charge amplifier and a digital oscilloscope for data acquisition. FPI induced mice were then immediately exposed to NBOT for 3 h. TBI injury without treatment group was kept in normal condition for 3 h. After 3h exposure to NBOT, animals were kept in a normal cage. After 24 hr, animal’s locomotor activity was assessed using IntelliCage system following FPI.

### Oxygen Therapy

Pure oxygen was delivered immediately after FPI. The mice were placed in a closed chamber to receive (97 ± 3%) O2 from the oxygen tank for 3h (47) under normal atmospheric pressure.

### Acclimatization period

Mice were housed in IntelliCage system (from TSE) (33 cm × 55 cm × 20 cm) containing standard sawdust bedding for four days of the initial acclimatization period. Equal number of mice belonging to each experiment ((i) Control (n=24), (ii) Sham group (n=24), (iii) (FPI) group (n=24) and (iv) FPI + NBOT group (n=24). Mice were allowed to freely access food and water throughout the acclimatization period and were housed under light-dark conditions (lights on: 08:00-20:00), an ambient temperature of 22 ± 10 C and 50%-60% relative humidity.

### Transponder implantation

After four days of the initial acclimatization period, all mice were implanted with micro transponders. Implantation was performed under light anesthesia and initiated by administering of Ketamine + Xylazine (1.6/0.10 mg per animal). The transponders were then subcutaneously injected into the scruff of the neck using the supplied disposable syringes and identification of each mouse was performed using a hand held electronic reader. The entire transponder implantation procedure lasted approximately 60min starting from initiation of anesthesia till complete recovery from anesthesia.

### IntelliCage apparatus and protocol

The IntelliCage system is fully automated, controlled by software operation from computer attached to the system (9). Briefly, IntelliCage is equipped with four corner chambers accessible through a ring antenna. In each corner, two doors controled the access to the two water bottles. Three events were recorded in the corners: 1) visit – each time a transponder is read by the circular antenna in conjunction with a presence heat sensor; 2) nose poke – each occasion an infrared beam, placed in front of the IntelliCage door is interrupted by animal; 3) lick – each time the signal from the —lick sensor || exceeds the threshold value, generally because of the animals’ touching the nipple of the water bottle. We have estimated the number of visits in each corner (as output by the Analyzer software), which is an indirect measure of activity of animals. IntelliCage software (versions 2.2.0 and 2.4.2) and IntelliCage corners (version 2.0) were used during the tests (http://www.newbehavior.com). After four days of habituation to the testing room, a transponder was subcutaneously injected into the scruff of the neck area of each mouse under Ketamine + Xylazine anesthesia. At least 24 h after transponder implantation, the mice were placed into the IntelliCage at the beginning of the dark phase.

### Behavior Protocol

**Figure 1:**
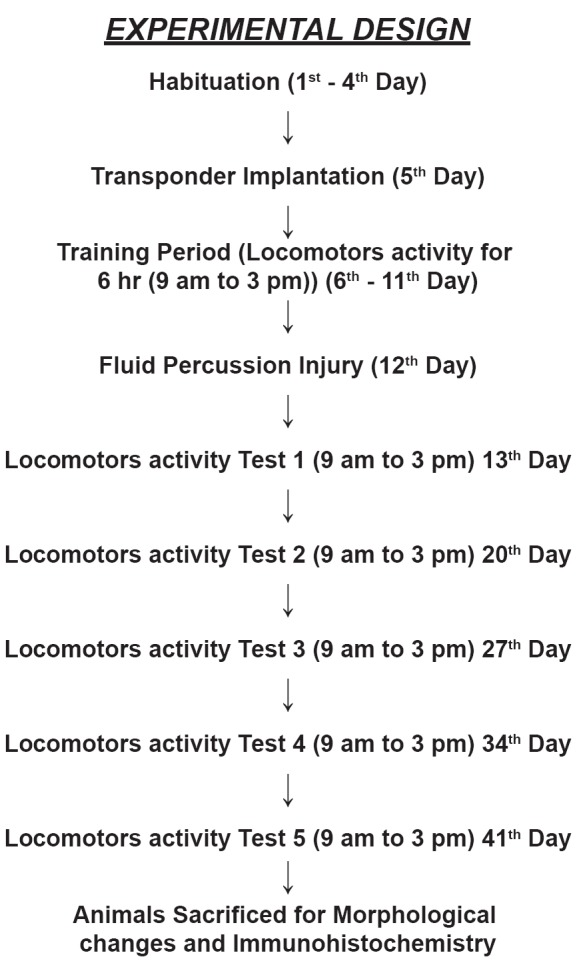
Experimental design of locomotor activity

After 24 hr of transponder implantation, animals were allowed to take water to all four corners for 5 days, from morning 9 am to 3 pm, which was considered as training for locomotor activity (in terms of number of visits in all four corners). Immediately after 5 days assessment of animal’s activity, FPI was performed in striatum of mice. Then animal’s locomotor activity were assessed on days 1, 7, 14, 21 and 28 days of following FPI (Figure 1).

### Tissue Processing

The animals were anaesthetized with Ketamine + Xylazine (2.6/0.16 mg/kg per animal) and were immediately perfused transcardially using ice-cold phosphate buffered saline (0.1 M, pH 7.4). Tissue sections were fixed using ice-cold 4% Para formaldehyde (dissolved in 0.1 M PBS pH 7.4). Then, the brains were removed from the cranium and fixed in same fixative for 24 h at room temperature.

### Histology

The six brains from in each group of mice were dehydrated and embedded with paraffin wax. Approximately 5μm coronal section was made. Sections were deparaffinized and rehydrated. Sections were stained using hematoxalin staining protocol (6). A drop of DPX was placed on the slide using a glass rod, avoiding the air bubbles. The DPX was allowed to spread beneath the cover slip, covering all the section. Slides were dried for overnight in the fume hood. Light microscopy was performed to evaluate the morphology of the right striatum of control, sham, FPI and FPI+Hyperoxia group. The small sized, dense, irregular shaped pyknotic cells were considered as dead. The photographs were captured in a compound light microscope attached with CCD camera. Images were imported into Adobe Photoshop 7.0 with a resolution of 300 dpi for correction of brightness and contrast.

### FITC-Annexin Apoptosis Assay

The sections from three brains in each group were deparaffinized and rehydrated according to Jingying *et al.*, 2010 (15). Then the sections were washed with PBS three times each five min. Annexin V-FITC was incubated was added to tissue sections for 1 h in dark place. The sections were observed under a fluorescence microscope using a dual filter set for FITC. Cells bound with Annexin V-FITC showed green staining in the plasma membrane. Cells that have lost membrane integrity showed red staining (PI) throughout the nucleus and green staining (FITC) on the cell surface (plasma membrane). Number of intact cells and number of dead cells were counted at 20× magnifications (15).

### Pan Neuronal Marking

The sections from each groups was deparaffinized and rehydrated by following protocol 2 × 10´ Xylene each, 2 × 5´ 100% ethanol, 1 × 3´ 95% ethanol, 1 × 3´ 80% ethanol, 1 × 5´ deionized H_2_O. Then sections were washed with ice-cold phosphate buffered saline (0.1 M, pH 7.4) 3 times for 5 min.Then sections were blocked with blocking buffer (1% BSA, 5% Serum, 0.2% Triton X in PBS for 1 hr at RT. After washing for 3 times, 5 min each, the sections were labelled with Milli-Mark fluoro Pan Neuronal marker (1:50) for 2 hr t RT (dilute antibody in blocking buffer). Washing was performed 3 × 5 min each and mounted with light diagnostics mounting fluid, and cover slip was placed on each slide. The staining was viewed using fluorescent microscope with FITC filter with 100× magnification (19).

### Immunohistochemistry

The sections from each groups was deparaffinized and rehydrated by following protocol Sun *et al*., 2009 (45). Sections were washed with PBS for 5min in three times. Then the sections were incubated with primary antibody D1 dopamine receptor (1:100) and D2 dopamine receptor (1:100) (dilute antibody in blocking buffer) for overnight. After overnight incubation, again sections were washed with PBS for 5 min each in three times. Then secondary antibody labelled with FITC 1:800) incubated for 2 hr at RT. Then washing was performed 3 × 5 min each and mounted with light diagnostics mounting fluid, and cover slip was placed on each slide. The staining was viewed using fluorescent microscope with FITC filter with 20× magnification (45).

### Statistics

Data were expressed as mean +/- standard error of the mean (SEM). Statistical analyses were performed with Prism 5 software (GraphPad, San Diego, CA) and Sigma Plot. Comparisons of means from four groups with repeated measure one-way ANOVA and Dunnet’s post hoc test (*p<0.05 compared to sham and FPI, ≠*p*<0.05 compared to FPI and FPI + Hyperoxia).

## RESULTS

### Fluid percussion injury in right striatum

**Figure 2:**
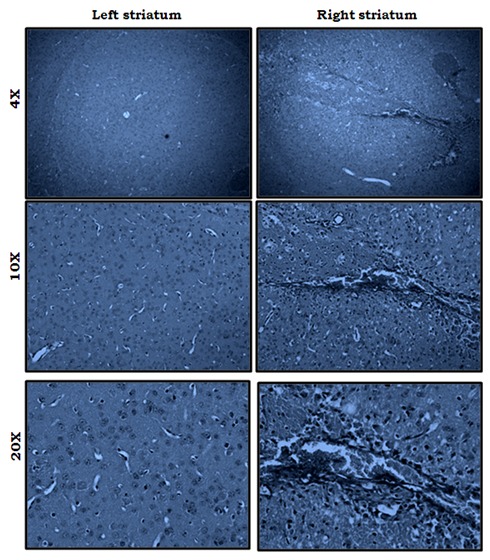
Shows comparion site between right and left striatum following fluid percussion injury

Figure 2 shows the damage site after induction of fluid percussion injury. The picture displays difference between left and right striatum of mice following closed traumatic brain injury. In other hand, it is considered as a confirmation for injury by fluid percussion device.

### Normabaric hyperoxia exposure on Locomotor Activity

**Figure 3:**
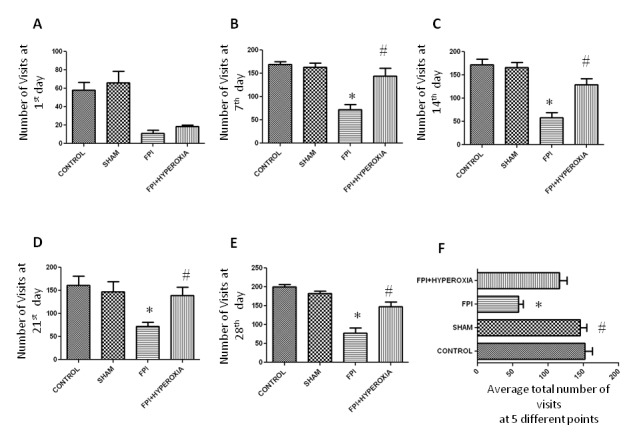
The graphs show the locomotor activity impairment after fluid-percussion injury. Data were expressed as mean +/- standard error of the mean (SEM). (*p<0.05 compared to sham and FPI, ≠p<0.05 compared to FPI and FPI+Hyperoxia)

Locomotor activity was assessed in long term up to 28 days at 7 days intervals following fluid percussion injury in striatum of mice. After induction of 1day, locomotor activity was assessed in IntelliCage for 6 hr. FPI animals with NBOT showed not significant (*p*>0.05) improvement in locomotor activity as compared to FPI (Figure 3A). FPI animals revealed significantly impairment in locomotor activity (in term of decreased number in visits of all four corner) as compared to control (*p*<0.05) and sham (*p*<0.05) group animals. There was no significant difference found between control and sham groups (*p*>0.05). Then animals were kept in normal cage until 7 day following FPI induction. On 7th day again locomotor activity was assessed for 6 hr in IntelliCage. Hyperoxia treated animals displayed significant (*p*<0.001) improvement as compared with FPI without treated group but no significant changes between control and sham (Figure 3B). Again animals were kept in normal cage for another 7 days. On 14th day the FPI animals significantly (*p*<0.005) showed impairment in locomotor activity as compared with control and sham. But FPI treated with hyperoxia was shown improvement as compared with FPI animals (Figure 3C). After keeping in normal cage for another 7 days, on day 21 , Hyperoxia treated animals showed significant (*p*<0.05) improvement as compared with FPI (Figure 3D). After assessing the locomotor activity, animals were kept in normal cage for 7 days, on day 28 again locomotor activity of mice assessed following FPI (Figure 3E). The results revealed that hyperoxia treatment animals showed significant improvement as compared with FPI without treated animals. There were no significant changes between control and sham. The average numbers of visits showed that hyperoxia animal’s locomotor activity improved as compared with FPI without treated animals (Figure 3F). There were no significant changes between control, sham and hyperoxia treated groups.

### Histology

**Figure 4:**
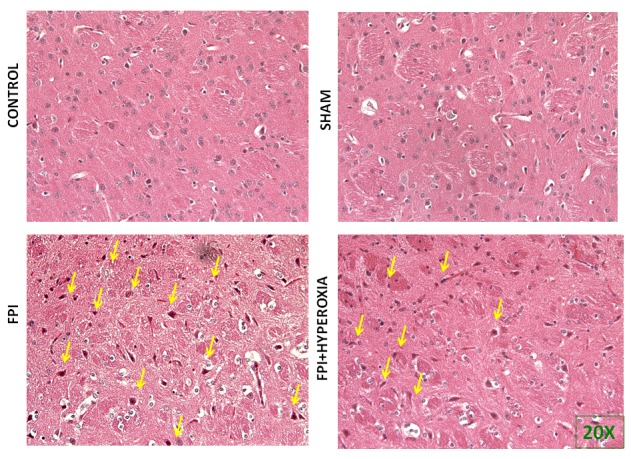
The figures show the morphological changes after fluid-percussion injury.

Following locomotor assessment, we have evaluated the morphological alterations in right striatum of mice. For assessing the morphological changes, we carried out haematoxylin and eosin staining. The results in Figure 4 have shown in 20× magnification to understand the morphological changes in point of injury. The images displayed the apparent neuronal damage with presence of pyknotic cells, nucleus shrinkage and irregular shaped cells in FPI group (Figure 4C) as compared with hyperoxia treated animals (Figure 4D). Whereas, the sham and control group animal did not show morphological changes in the right striatum (Figure 4A & 4B). The results also found that the eurons were shrunken apparently, and pyknotic in nature.A few of them also exhibited irregular shape and tangle like appearance and yet others were large dot like in outline. This suggested that the post trauma stage following FPI showed the more dead cells as compared to NBO exposure group.

### Apoptotic cell death in right striatum

**Figure 5:**
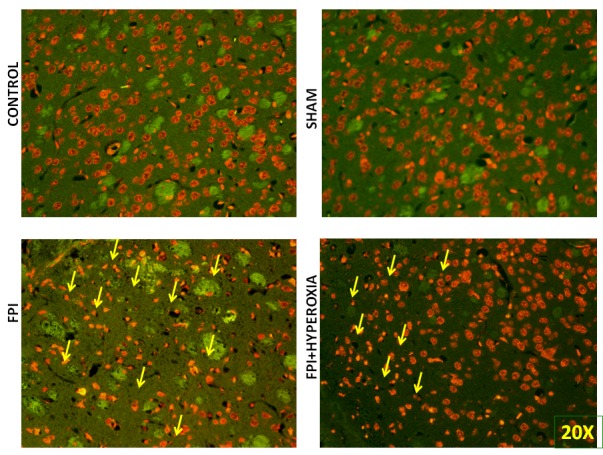
The figures suggest that fluid-percussion injury leads to neuronal damage (FITC -TUNAL ASSAY)

To understand the mechanisms involved in impairment of locomotor activity following FPI, apoptotic cell death assay was carried out to know role of neuronal cell death following induction of FPI on right striatum of mice. We have determined the cell death at day 28 of post FPI. The FPI group proved significant cell death as compared to control and shamp group (Figure 5A & 5B), Whereas FPI with NBO treated group mice showed less neuronal damage as compared with FPI without NBO treated group (Figure 5C & 5D). Morphological characteristics of apoptotic cell death showed by nuclear shrinkage, condensed cytoplasm and presence of apoptotic bodies. Counting of dead cells and live was represented in Graph 1, showing significant reduction in cell death in NBOT group as compared to FPI. To further confirm neuronal damage, we have performed pan neuronal staining to evaluate the structural changes in striatum of mice following FPI.

### Pan Neuronal Marking

**Figure 6:**
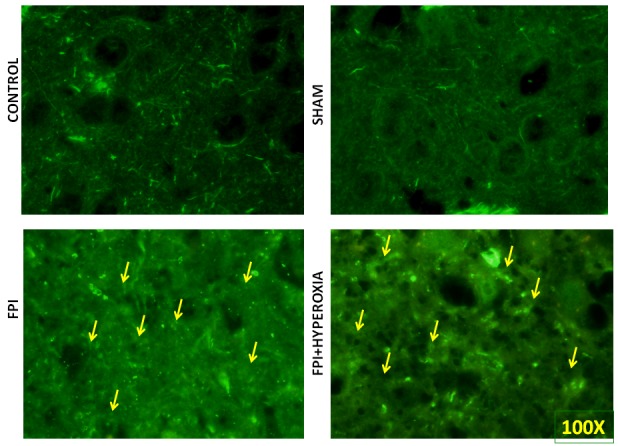
The figures show the pan neuronal marking for complete neuronal structure. The results suggested that neuronal structure has been impaired after fluid-percussion injury

For further identification of alterations in complete neuronal structure, we have defined the changes in axonal length, soma and spines by pan neuronal marking using neuronal marker Alexa 488. The result has revealed that after 28 days of FPI, the neuronal structure of soma, axon and spine has been distorted as compared to sham and control group (Figure 6A & 6B). Neuronal structural changes were qualitatively measured in axonal length, irregular shaped soma and distorted spines in FPI group as compared to NBOT group (Figure 6C & 6D). The neuronal damage was found to be severe in striatal neurons at 28th day following FPI as compared to NBOT animals.

### Immunohistochemistry

**Figure 7:**
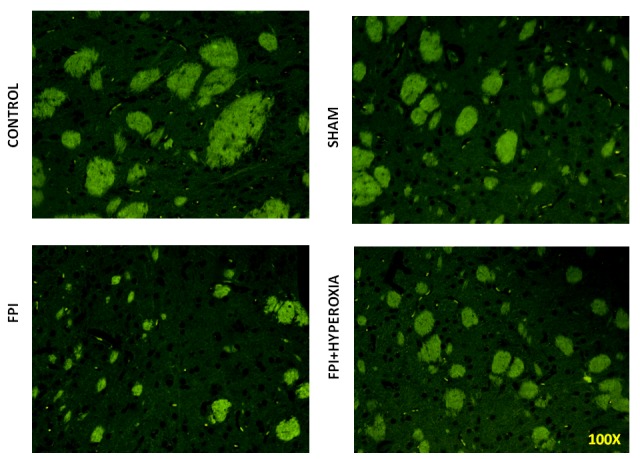
The figures show the expression of D1 dopamine receptor in right striatum

**Figure 8:**
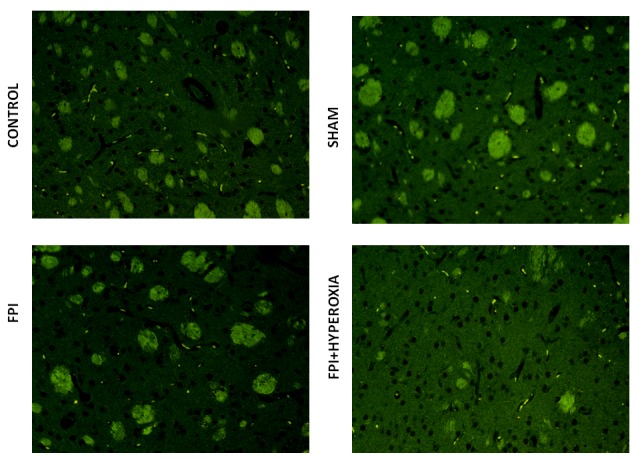
The results demonstrate the expression of D2 dopamine receptor in right striatum of mice

**Figure 9:**
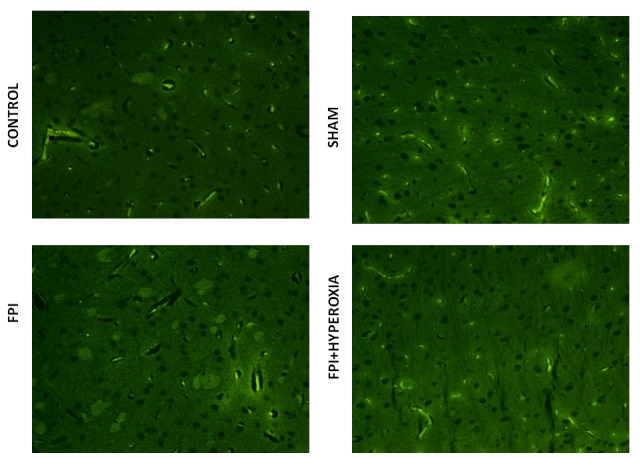
The figures suggested that the expression of DAT expression in right striatum of mice

**Figure 10:**
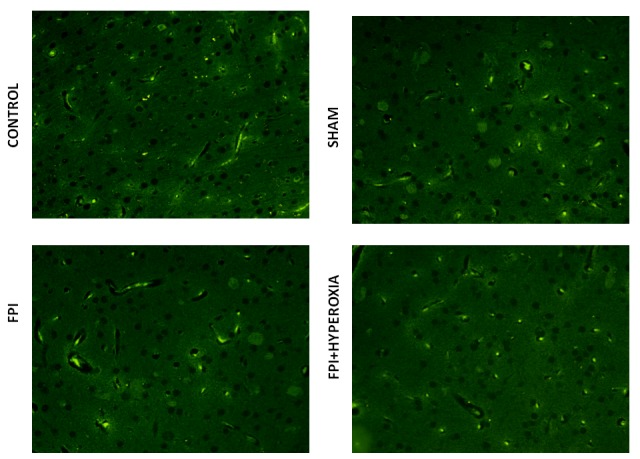
The pictures show the expression of VMAT expression in right striatum

We wanted to know the role of dopamine receptors in locomotor activity of mice followingFPI. For that DR1, DR2, DAT and VMAT dopamine genes has been analysised in striatum by using FITC conjugated secondary antibody. The results revealed that FPI group shows the decreased expression of DR1, DR2, DAT and VMAT in striatum as compared to control and sham group of animals. Immediate exposure to NBOT animals shows the increased expression of DR1 (Fig. 7), DR2 (Fig. 8), DAT (Fig. 9) and VMAT (Fig. 10) dopamine receptors in striatum following FPI. DR1, DR2, DAT and VMAT expression less in NBOT treated animals as compared to control and sham group. There were no changes found in control and sham groups of animals.

## DISCUSSION

In the present study to aimed to investigate the role of normabaric hyperoxia treatment following FPI. In results, following fluid percussion injury (Figure 2) displayed the comparision between right and left striatum of mice then the locomotor activity of mice has been assessed at 7 days interval for 28th days. The locomotor activity was affected because of the induction of fluid-percussion injury in the striatum. This impairment in locomotor activity because of and edema which might be caused by fluid-percussion injury (35, 39). These impacts may cause primary damage in right striatum of mice. Ultimately, this leads to hypoxic-ischemic injury as a result of inadequate oxygen supply to the brain tissue. As result, brain tissue oxygen tension would be increased (38). In our current experiment, the results suggested that locomotor activity of mice were gradually improved in Normabaric hyperoxia treated animals as compared to injured animals without treatment. Locomotor activity of mice were improved in treated animals because of normobaric hyperoxia increased tissue O2 delivery in striatum (41). Even though, many mechanisms are involved to determine the locomotive activity of mice following FPI in striatum, we evaluated the dopamine system associated with locomotive behavior.

In the present study, we investigated the dopamine genes such as D1 and D2 receptors, DAT and VMAT transporters after assessing behavior of mice. Dopamine (DA) is well-recognized for its determinant role in the modulation of various brain functions. DA was also found in locomotor activity in vertebrate and in vertebrate (20). The D1 subtype is the most abundant dopamine receptor in the CNS. This G-protein-coupled receptor stimulates adenylyl cyclase and activates cyclic AMP-dependent protein kinases. D1 receptors regulate neuronal growth and development, mediate some behavioral responses, and modulate dopamine receptor D2-mediated events (31). The D2 subtype of the dopamine receptor. Several pharmacological treatments aimed at the dopamine system have been reported to be beneficial in improving cognitive functions in animals and humans. Catecholamine agonist therapy shows motor and cognitive improvement in humans and animals (33). Methylphenidate (16) and d-amphetamine (10), which increase synaptic dopamine levels by inhibiting dopamine transporter (DAT) function, have shown to enhance functional outcome after experimental TBI. Research has been carried out to find solutions to improve the dopamine system following TBI.

The striatum represents the main input nucleus of the basal ganglia, a system of subcortical nuclei critically involved in motor control and motivational processes and altered in several conditions such as Parkinson’s and Huntington’s diseases or drug addiction and schizophrenia (18, 29). In the present study, the results suggested that following FPI, the dopaminergic genes are down regulated. There are four genes of dopamine has been studied in this experiment such as D1 and D2 receptors, DAT transporter and VMAT1. In results, we found that all dopaminergic system has been down regulated. Previous reports suggested that changes in dopaminergic signaling system after a TBI insult include initial increase in D1 like DA receptors, but decrease in D1 and D2 like DA receptors and DA transporter. Dopamine transporter protein expression was decreased in the injured right striatum at 28 days as compared with sham control rats. The decrease in dopamine transporter protein levels may reflect a traumatic brain-injury-induced down-regulation of dopamine transporter and a loss of dopaminergic fibers. Another study also supported that DAT decreases chronically after TBI. Impaired MAT function/activity may contribute to symptoms of depression, anxiety, restless leg syndrome, akathisia, Parkinson’s disease, social anxiety, and many other conditions, via inhibition of normal dopamine release into the synapse. Besides, In the mammalian CNS the dopamine transporter (DAT) is the primary mechanism for clearance of dopamine from the extracellular space.

**Figure 11:**
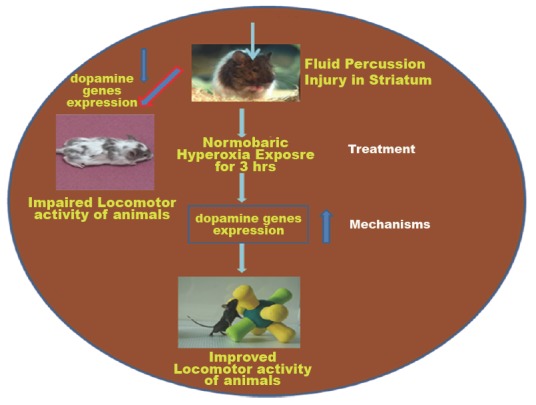
The Schematic diagram shows that Normabaric hyperoxia could improve locomotor activity of mice following fluid-percussion injury

The transporter thereby serves as an optimal target to regulate dopamine neurotransmission. In a single photon emission tomography (SPET) study conducted in TBI patients, it is observed that striatal DAT binding was significantly reduced (8) and DAT can be regulated by the level of DA available in synaptic space. Reduced activity of DAT resulted in motor abnormalities involving hyperactivity as has been observed in DAT knockout mice (11). While D1 knock out mouse showed spatial memory impairment Perona *et al*., 2008 (32) as well as spatial learning deficits. D2 dopamine receptors are seen to be highly expressed in the striatum compared to other brain regions and polymorphism in genes modulating D2 dopamine receptor results in bad performance in response latency for subjects with TBI (24). Since the dopamine transporter, dopamine receptor D1 and D2 have higher cellular distributions in striatum regions, damage to the striatal basal ganglia resulted in persistent cognitive dysfunctions such as impaired processing speed, attention and memory deficit as well as motor difficulties in initiating movement, sustaining movement and stopping movement (Figure 11).

## CONCLUSION

Closed traumatic brain injury in striatum could cause impairment in locomotors acitivity. In addtion, dopamine system has been altered through this damage. Dopaminergic neruons is most responsible for locomotors activity of animals as well as human benings. Dopaminergic neurona damage could be possible reasons for movement disorder or motor difficulties following TBI. Immediate exposure to NBOT could be possible therapeutic for closed traumatic brain injury which could improve the dopaminergic system and enhanced locomotor activity.
